# Influence of different elicitors on BIA production in *Macleaya cordata*

**DOI:** 10.1038/s41598-020-79802-0

**Published:** 2021-01-12

**Authors:** Peng Huang, Liqiong Xia, Li Zhou, Wei Liu, Peng Wang, Zhixing Qing, Jianguo Zeng

**Affiliations:** 1 Hunan Key Laboratory of Traditional Chinese Veterinary Medicine, Hunan Agricultural University, Changsha, 410128 Hunan China; 2 Animal Nutritional Genome and Germplasm Innovation Research Center, College of Animal Science and Technology, Hunan Agricultural University, Changsha, 410125 Hunan China; 3 Clinical Pharmacy, Yueyang Hospital of TCM, Yueyang, 414000 Hunan China; 4 College of Veterinary Medicine, Hunan Agricultural University, Changsha, 410128 Hunan China; 5 College of Food Science and Technology, Hunan Agricultural University, Changsha, China; 6 National and Local Union Engineering Research Center of Veterinary Herbal Medicine Resource and Initiative, Hunan Agricultural University, Changsha, 410128 Hunan China

**Keywords:** Plant stress responses, Secondary metabolism

## Abstract

Sanguinarine (SAN) and chelerythrine (CHE) have been widely used as substitutes for antibiotics for decades. For a long time, SAN and CHE have been extracted from mainly *Macleaya cordata*, a plant species that is a traditional herb in China and belongs to the Papaveraceae family. However, with the sharp increase in demand for SAN and CHE, it is necessary to develop a new method to enhance the supply of raw materials. Here, we used methyl jasmonate (MJ), salicylic acid (SA) and wounding alone and in combination to stimulate aseptic seedlings of *M. cordata* at 0 h, 24 h, 72 h and 120 h and then compared the differences in metabolic profiles and gene expression. Ultimately, we found that the effect of using MJ alone was the best treatment, with the contents of SAN and CHE increasing by 10- and 14-fold, respectively. However, the increased SAN and CHE contents in response to combined wounding and MJ were less than those for induced by the treatment with MJ alone. Additionally, after MJ treatment, SAN and CHE biosynthetic pathway genes, such as those encoding the protopine 6-hydroxylase and dihydrobenzophenanthridine oxidase enzymes, were highly expressed, which is consistent with the accumulation of SAN and CHE. At the same time, we have also studied the changes in the content of synthetic intermediates of SAN and CHE after elicitor induction. This study is the first systematic research report about using elicitors to increase the SAN and CHE in *Macleaya cordata*.

## Introduction

The benzophenanthridine alkaloids (BIAs) are a large and diverse alkaloid group, and these compounds, such as sanguinarine (SAN), chelerythrine (CHE), protopine (PRO), and allocryptopine (ALL), exhibit a wide range of biological activities^1^. SAN has a wide spectrum of biological activities, including strong antitumour^2^, antimicrobial^3^ and anti-inflammatory activities^4^. In addition, SAN and CHE are as natural growth promoters that can be used as alternatives to antibiotic growth promoters in the livestock industry^5^. In addition, SAN has real potential as an effective antischistosomal drug^6^. Both PRO and ALL were demonstrated to have anti-bacterial, anti-viral, anti-fungal and anti-parasitic effects^7–10^. PRO has potential uses as a neuroprotective agent in stroke and as an antidepressant for mood disorders^11^. Currently, SAN and CHE are extracted from mainly *Macleaya cordata*, a plant species that is a traditional herb in China and belongs to the Papaveraceae family^5,12^. In 2006, with the ban of using low-dose antibiotics as growth promoters added in animal feed in the European Union, natural growth promoters (NGPs), such as phytogenics, are extensively exploited as alternatives to antibiotics in livestock production^13^. Currently, the annual requirement for this plant is increasing daily due to its commercial value. Unfortunately, this plant has generally been collected from the wild for SAN commercial production. Therefore, the massive collection of wild resources of *M. cordata* caused a decline in its population and we need to establish a new stable supply method and develop a viable alternative to SAN production.

Plant tissue culture has become a promising alternative strategy for the sustainable and industrial-scale production of secondary metabolites. There are multiple advantages of medicinal plant tissue culture. (i) It is not affected by environmental or geographic conditions. (ii) It can strictly control production and quality and (iii) shorten the growth cycle compared to that of the intact plant. (iv) It avoids taking land resources. In fact, some plant secondary metabolites have been produced using this method, such as shikonin^14,15^, ginseng saponins^16^ and paclitaxel^17–19^, over the past decades. Based on this strategy, some elicitors have been widely used to enhance secondary metabolite production in many plant species. For example, methyl jasmonate (MJ) treatments were found to enhance the production of camptothecin in *Ophiorrhiza mungos*^20^ and the content of olide A, withanolide A, withanone, and withaferin A in *Withania somnifera*^21^. In addition, salicylic acid (SA) can increase the accumulation of secondary metabolites in *Salvia miltiorrhiza* cells^22^ and *Daucus carota*^23^. Moreover, wounding responses have always occurred. For example, wind, hail, sand and cuts can increase the secondary metabolite production of plants^24,25^. In fact, some researchers have to enhance SAN in *Argemone Mexicana* by MJ, *Fusarium oxysporum* homogenates^26^. Additionally, the sequential application of MJ, SA and YE also can increase the SAN content over 9 times in *A. Mexicana*^27^. Although the continuous treatment of MJ, SA and YE also can increase SAN 5.5 times in *Eschscholtzia californica*^28^. However, another research found that the effect of MJ treatment was better^29^. In addition, manganese chloride is also used to try to increase BIAs in *E. californica*^30^. However, there still is no report available regarding the elicitor or mechanical damage effect on BIA production in the tissue culture of *M. cordata.*

**Figure 1:**
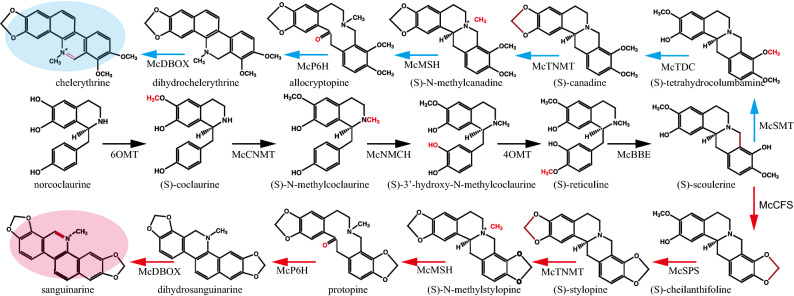
The metabolic pathway of sanguinarine and chelerythrine. 6OMT, norcoclaurine 6′-*O*-methyltransferase; CNMT, coclaurine-*N*-methyltransferase; NMCH, *N*-methylcoclaurine hydroxylase; 4OMT, 4′-*O*-methyltransferase; BBE, berberine bridge enzyme; CFS, cheilanthifoline synthase; SPS, stylopine synthase; TNMT, tetrahydroprotoberberine cis-*N*-methyltransferase; MSH, (*S*)-cis-*N*-methylstylopine 14′-hydroxylase, P6H, protopine 6′-hydroxylase; DBOX, dihydrobenzophenanthridine oxidase; TDC, (*S*)-canadine synthase; SMT, (*S*)-scoulerine 9′-*O*-methyltransferase.

In 2017, the whole genome of *M. cordata* was sequenced, which is the first species in Papaveraceae to have a completed genome-wide sequence^5^. Research on elicitors of *M. cordata* will further promote the study of the regulation of SAN synthesis. Currently, the protocol of regeneration and transformation in *M. cordata* has been established, and the SAN biosynthetic pathway and genes in *M. cordata* have been validated (Fig. 1)^5,31–33^. The aim of this study was to evaluate the effects of MJ, SA and wounding on BIA metabolism in plant tissue culture seedlings to enhance SAN production in *M. cordata*. Moreover, multiple processing at different times was evaluated. Additionally, the effect of different elicitors was evaluated on the basis of the metabolic profile and gene expression in the SAN and CHE biosynthesis pathway.

## Materials and methods

### Plant materials and treatments

Sixty-day-old lines of *M. cordata* were maintained in hormone-free solid medium. All the cultures were under a 16/8 h (light/dark) cycle (4500 to 9000 lx) until used for transformation, and these tissues were used for all experiments. MJ (≥ 98%) and SA (≥ 99%) were purchased from Sigma (USA). They were filter sterilized using 0.22 μM membrane filters (Millipore, USA) and diluted in DMSO (dimethyl sulfoxide). For wounding treatment, 60-day-old *M. cordata* seedlings were wounded in the leaves of the seedlings using sterile needles and placed in Murashige and Skoog (MS) solid medium. For the combined treatment, seedlings were first wounded and then placed in MS containing 100 μM MJ or 100 μM SA. The samples of the control group were also placed in MS medium without any treatments. All the samples were grown at 25 °C under a 16 h photoperiod. For gene expression and metabolic profile studies, samples treated as described above were harvested at different time points. Finally, all samples were immediately frozen in liquid nitrogen and stored at − 70 °C for future experiments. All the samples were collected at 0 h, 24 h, 72 h and 120 h to test the optimum incubation time required for the treatment to evoke the maximum response. Samples were collected in triplicate each time.

### Metabolite extraction from *M. cordata* and LC/triple-quadrupole (QQQ) MS analysis

The methods of alkaloid extraction and metabolic analysis were performed using previously described methods^5^. The freeze-dried plant tissues (0.5 mg) were mixed with methanol (25 mL). Then, the samples were extracted for 30 min by ultrasonication, centrifuged at 14,000 rpm for 15 min and filtered through a 0.22-mm membrane filter. Finally, the solution was chromatographically separated by an ultra-HPLC Agilent 1290 instrument using a BEH C18 column. The automatic sampler temperature was set at 6 °C. Ultra-High-Performance Liquid Chromatography (UHPLC) was coupled with a QQQ mass spectrometer (6460A, Agilent). A calibration curve was generated using 5 points and was used to evaluate the absolute quantification of the target compound.

### RNA extraction and qRT-PCR analyses

Control and treated (elicitors and wounding) seedling tissues (100 mg) were collected at the different time points (0 h, 24 h, 72 h and 120 h) for RNA extraction. All tissues were ground in liquid nitrogen, and total RNA was extracted with an RNA extraction kit (TaKaRa, MiniBEST Plant Genomic DNA, China) according to the manufacturer’s instructions. We used PrimeScript™RT Master Mix (TaKaRa, China) to synthesize cDNA according to the manufacturer’s instructions. The primers used for gene expression by qPCR are listed in Table 1. We performed quantitative PCR (qPCR) using an ABI 7300 and SYBR Premix (Roche, Switzerland) according to the manufacturer’s instructions. The 18S gene was applied as the housekeeping gene in all applications. The relative gene expression was calculated by the formula 2^−ΔΔCt^.

**Table 1:** Nucleotide sequences of primers.

Primer Name	Oligonucleotide Sequences (5′–3′)
*McP6H*-QP-F	CATCAAGGACGTTCGAGCCT
*McP6H*-QP-R	CTCCTCACCACGCACAATCT
*McDBOX*-QP-F	ACTGTTGCCACGGTCGATAG
*McDBOX*-QP-R	TGGAGGAGCTTGTCAACACC
*18S*-QP-F	CTTCGGGATCGGAGTAATGA
*18S*-QP-R	GCGGAGTCCTAGAAGCAACA

### Statistical analysis

All the experiments were performed in triplicates. Data were recorded at four different time points (0 h, 24 h, 72 h and 120 h). We used a one-way ANOVA with GraphPad Prism software (Version 8.4.0), followed by the Tukey’s honestly significant difference (HSD) post hoc test for mean comparison.

## Results

### Effect of different elicitors on SAN and CHE, ALL, PRO, dihydrosanguinarine (DHSAN) and dihydrochelerythrine (DHCHE) production

**Figure 2:**
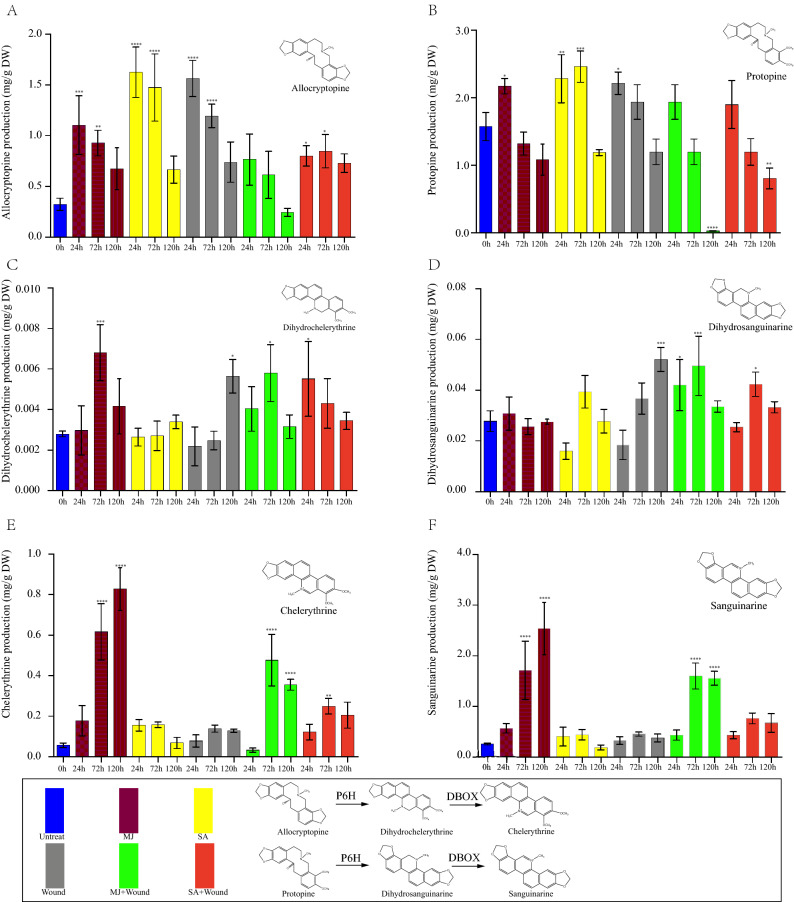
Time course analysis of alkaloid levels in different treatment groups. (**A**) Allocryptopine. (**B**) Protopine. (**C**) Dihydrochelerythrine. (**D**) Dihydrosanguinarine. (**E**) Chelerythrine. (**F**) Sanguinarine. Asterisks denote significant changes (Tukey’s test, * indicates *P* < 0.05, ** indicates *P* < 0.01, *** indicates *P* < 0.005, and **** indicates *P* < 0.001) compared with the 0 h group.

In *M. cordata*, PRO and ALL were transformed into 6-hydroxyprotopine by P6H and into the spontaneous intramolecular rearrangement forms DHSAN and DHCHE^34,35^, and these molecules were finally were oxidized to SAN and CHE by DBOX^36,37^. The contents of SAN and CHE were significantly higher after the MJ treatment than after the other treatments (*P* < 0.05) at 120 h (Fig. 2E,F). Compared with those in the untreated group, the contents of SAN and CHE were increased by 10- and 14-fold, respectively (Fig. 2E,F). In addition, treatment with SA and wounding resulted in less SAN and CHE accumulation compared with that in the MJ group (Fig. 2E,F). However, combined MJ and wounding enhanced SAN, CHE, PRO, and ALL production (Fig. 2A,B,E,F). Additionally, we compared the changes in alkaloid content after treatment for 120 h. The results showed that the impact of MJ increased with time. The SAN and CHE production showed a maximum of 2.54 ± 0.42 mg/g at 120 h after initiation of MJ treatment, and the combined MJ and wounding treatment produced a maximum of 1.6 ± 0.25 mg/g at 72 h after treatment, which was lower than that after the treatment with MJ alone. Finally, we found that 3 treatments (MJ, SA, and wounding) had significant effects on the PRO and ALL contents (Fig. 2). In contrast, the PRO and ALL contents continuously declined over time and were lower than those in the untreated group at 120 h. In addition, treatment with MJ significantly increased (*P* < 0.05) the DHCHE content at 72 h. However, the wounding treatment in combination with either of the two other treatments (MJ or SA) had greater effects than the control, MJ and SA groups on the DHSAN content (Fig. 2).

### Effect of different elicitors on P6H, DBOX genes expression levels

**Figure 3:**
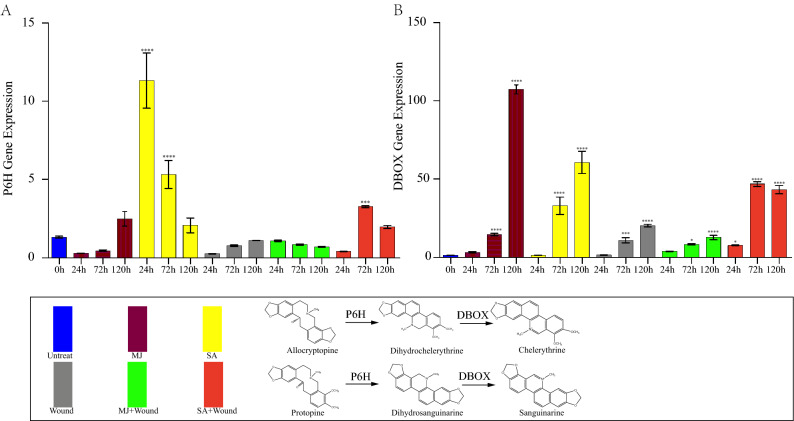
The time course analysis of gene expression in different treatment groups. (**A**) Protopine 6-hydroxylase (P6H). (**B**) Dihydrobenzophenanthridine oxidase (DBOX). Asterisks denote the significant changes (Tukey’s test, * means *P* < 0.05, ** means *P* < 0.01, *** means *P* < 0.005, **** means *P* < 0.001) compared with 0 h group.

The production of protopine and sanguinarine need to be catalysed by the enzymes encoded by the P6H and DBOX genes; therefore, the effect of the induction mode on the content can be further analysed by detecting the expression levels of these two genes. By analysing the mRNA expression levels of the P6H and DBOX genes (Fig. 3), which catalyse production of protopine and sanguinarine, the effect of the elicitor can be further analysed. We found that the P6H gene expression was the highest in the SA group at 24 h, which then decreased with time. In contrast to that in the SA group, the expression level of the P6H gene was highest in the SA + wounding group at 72 h. Interestingly, the expression pattern of the DBOX gene was the opposite of that of P6H. All the treatments increased the expression of the DBOX gene, which increased with time, except for that in the SA + wounding group at 120 h. The highest expression was induced by MJ treatment in all methods, followed by that induced by SA treatment.

## Discussion

Elicitation is one of the most effective methods to enhance the accumulation of secondary metabolites in plants^38–42^. Some biotic elicitors or physical stimulations are always used to improve the production of secondary metabolites in vitro ^43^. MJ and SA have been proven to be signalling molecules involved in the plant defence response and are now widely used as elicitors for secondary metabolite production in vitro^44–46^. Additionally, the concentration, type and time course of elicitors have a great impact on the accumulation of secondary metabolites ^47^. In previous studies, the treatment of plant cultures with MJ has been shown to increase the accumulation of triterpenoid saponins^48^, alkaloids^27,28,49^, and ginsenoside^50^. Similarly, many studies have found that secondary metabolite products in SA-treated plant cells are also significantly increased^51–53^. In addition, studies have found that mechanical damage increases and induces the production of various secondary metabolites, including volatiles^54–56^. Wounding treatment also increased the accumulation of volatiles, including monoterpenes and sesquiterpenes^57^. In *A. mexicana* suspensions, there have been some inconsistencies regarding the content of SAN after treatment with SA and MJ^27,49^. However, in California poppy, both MJ and SA significantly increased (*P* < 0.05) the content of SAN^28,29^. Consistent with these findings, we observed an enhancement of SAN content in the MJ treatment group in *M. cordata*. Interestingly, SAN content decreased in the MJ + wounding or SA + wounding groups, and the same phenomenon also appeared in *Panax ginseng*^57^, in which necrosis caused by mechanical wounding at damaged sites can be recovered by exogenously supplied MJ. The biosynthesis of many secondary metabolites is mainly regulated by gene expression levels. With the completion of the whole genome sequencing of *M. cordata*, some key genes involved in biosynthesis of sanguinarine have been identified^5^, such as P6H and DBOX. P6H is responsible for catalyzing PRO and ALL to produce SAN and CHE, respectively. DBOX is the key gene that catalyzes the last step of SAN and CHE synthesis. Based on the gene expression data, elicitors have different effects on the P6H and DBOX genes. Specifically, MJ has a greater effect on the DBOX gene than on P6H. In contrast, SA has a greater effect on the P6H gene than on DBOX. In *Argemone mexicana*, yeast extract increases DBOX activity, but does not increase after MeJa induction^58^. This shows that different species are affected differently by the elicitor. Based on these results, we believe that MJ is the best elicitor for increased SAN production, with hope for its application to the production of sanguinarine during harvesting in the future.
